# RANKL, but Not R-Spondins, Is Involved in Vascular Smooth Muscle Cell Calcification through LGR4 Interaction

**DOI:** 10.3390/ijms25115735

**Published:** 2024-05-24

**Authors:** Sara Fernández-Villabrille, Julia Martín-Vírgala, Beatriz Martín-Carro, Francisco Baena-Huerta, Nerea González-García, Helena Gil-Peña, Minerva Rodríguez-García, Jesús María Fernández-Gómez, José Luis Fernández-Martín, Cristina Alonso-Montes, Manuel Naves-Díaz, Natalia Carrillo-López, Sara Panizo

**Affiliations:** 1 Metabolismo Óseo, Vascular y Enfermedades Inflamatorias Crónicas, Instituto de Investigación Sanitaria del Principado de Asturias (ISPA), 33011 Oviedo, Spain; 2 Redes de Investigación Cooperativa Orientadas a Resultados en Salud (RICORS), RICORS2040 (Kidney Disease), 33011 Oviedo, Spain; 3 AGC de la Infancia y Adolescencia, Hospital Universitario Central de Asturias (HUCA), Instituto de Investigación Sanitaria del Princiado de Asturias (ISPA), 33011 Oviedo, Spain; 4 Bone and Mineral Research Unit, Hospital Universitario Central de Asturias, 33011 Oviedo, Spain; 5 Urology Unit, Hospital Universitario Central de Asturias, 33011 Oviedo, Spain; 6 Department of Medicine, Universidad de Oviedo, 33006 Oviedo, Spain

**Keywords:** LGR4, RANKL, R-spondins, vascular calcification, phosphorus

## Abstract

Vascular calcification has a global health impact that is closely linked to bone loss. The Receptor Activator of Nuclear Factor Kappa B (RANK)/RANK ligand (RANKL)/osteoprotegerin (OPG) system, fundamental for bone metabolism, also plays an important role in vascular calcification. The Leucine-rich repeat-containing G-protein-coupled receptor 4 (LGR4), a novel receptor for RANKL, regulates bone remodeling, and it appears to be involved in vascular calcification. Besides RANKL, LGR4 interacts with R-spondins (RSPOs), which are known for their roles in bone but are less understood in vascular calcification. Studies were conducted in rats with chronic renal failure fed normal or high phosphorus diets for 18 weeks, with and without control of circulating parathormone (PTH) levels, resulting in different degrees of aortic calcification. Additionally, vascular smooth muscle cells (VSMCs) were cultured under non-calcifying (1 mM phosphate) and calcifying (3 mM phosphate) media with different concentrations of PTH. To explore the role of RANKL in VSMC calcification, increasing concentrations of soluble RANKL were added to non-calcifying and calcifying media. The effects mediated by RANKL binding to its receptor LGR4 were investigated by silencing the LGR4 receptor in VSMCs. Furthermore, the gene expression of the RANK/RANKL/OPG system and the ligands of LGR4 was assessed in human epigastric arteries obtained from kidney transplant recipients with calcification scores (Kauppila Index). Increased aortic calcium in rats coincided with elevated systolic blood pressure, upregulated *Lgr4* and *Rankl* gene expression, downregulated *Opg* gene expression, and higher serum RANKL/OPG ratio without changes in *Rspos* gene expression. Elevated phosphate in vitro increased calcium content and expression of *Rankl* and *Lgr4* while reducing *Opg*. Elevated PTH in the presence of high phosphate exacerbated the increase in calcium content. No changes in *Rspos* were observed under the conditions employed. The addition of soluble RANKL to VSMCs induced genotypic differentiation and calcification, partly prevented by LGR4 silencing. In the epigastric arteries of individuals presenting vascular calcification, the gene expression of *RANKL* was higher. While RSPOs show minimal impact on VSMC calcification, RANKL, interacting with LGR4, drives osteogenic differentiation in VSMCs, unveiling a novel mechanism beyond RANKL-RANK binding.

## 1. Introduction

The abnormalities in the bone–vascular axis that occur during the progression of chronic kidney disease (CKD) arise from a complex interplay of several factors. These factors include age, the extent of renal damage, elevated phosphate and fibroblast growth factor 23 (FGF23) levels, and reductions in renal klotho, calcitriol, and calcium (Ca) serum levels [1,2,3,4]. Additionally, hyperphosphatemia stimulates the production of parathyroid hormone (PTH), which is the primary regulator of the Receptor Activator of Nuclear Factor kappa B (RANK)/RANK ligand (RANKL)/osteoprotegerin (OPG) system [5,6,7]. This pathway is pivotal in controlling both bone turnover and vascular calcification.

Recently, leucine-rich repeat-containing G-protein-coupled receptor 4 (LGR4) has been identified as a new receptor for RANKL [8]. LGR4 contributes to bone formation [9] by enhancing osteoblast maturation and mineralization and by counteracting the RANKL-driven osteoclastogenesis [8]. Indeed, vascular smooth muscle cells (VSMCs) in the middle layer of arteries share a mesenchymal origin with osteoblasts and can undergo phenotypic transformations under specific conditions, acquiring osteoblast-like properties that lead to vascular calcification. Given the role of LGR4 in osteoblast differentiation, it is reasonable to speculate that it could be involved in the differentiation of VSMCs into osteoblast-like cells, thereby affecting vascular calcification. In the vasculature, the increase in RANKL and the decrease in OPG are known to promote vascular calcification [10,11], and recent evidence suggests that these changes are accompanied by an upregulation of LGR4 expression [12]. In fact, LGR4 has been detected in the calcified areas of the arteries from uremic rats. Furthermore, in vitro studies have shown that the deletion of the *Lgr4* gene in VSMCs prevents calcification induced by high phosphate and PTH levels [12].

Moreover, LGR4 has been identified as a second-class receptor for the R-spondins (RSPOs), a family of four secreted proteins that have emerged as important activators of the Wnt signaling pathway, which is essential for normal bone formation [13,14] and may also play a role in vascular calcification [15,16,17,18]. Numerous studies have described the involvement of various RSPOs in bone remodeling by regulating osteogenic genes [19,20]. However, their potential role in the process of vascular calcification remains unknown.

Therefore, the main aim of this study was to better understand the mechanism by which LGR4 contributes to vascular calcification and to explore the role of its ligands, RSPOs and RANKL, in this process.

## 2. Results

### 2.1. Rat Experimental Study

#### 2.1.1. Biochemical and Renal Function Parameters

All groups of subtotal nephrectomized (NX) rats showed a significant reduction in creatinine clearance. Furthermore, among the NX rats, those on a high phosphorus (HP) diet displayed the highest serum phosphate levels, with the microsurgical parathyroidectomy (PTX) PTX NX HP rats having significantly higher levels (Table 1).

PTH (1–84) serum levels increased in NX rats, especially in the NX HP group. In rats that had undergone parathyroidectomy (PTX NX groups), serum levels of PTH (1–84) were undetectable, and the subcutaneous PTH (1–34) pellet was sufficient to maintain serum PTH (1–34) levels within a comparable range to that observed in the Sham-operated group. Although the PTH pellet was effective in sustaining serum Ca levels in the group fed a normal phosphorus (NP) diet (NX PTX NP), it failed to do so in the group fed an HP diet (NX PTX HP). Serum PTH (1–34) levels were higher in the NX HP rats. All nephrectomized groups, except PTX NX HP, exhibited significant increases in serum FGF23 levels compared to the Sham-operated groups. Elevated phosphorus intake significantly increased serum calcitriol in the Sham HP and NX HP groups but not in the PTX NX HP group (Table 1).

#### 2.1.2. Aortic Ca Content and Arterial Blood Pressure

**Figure 1:**
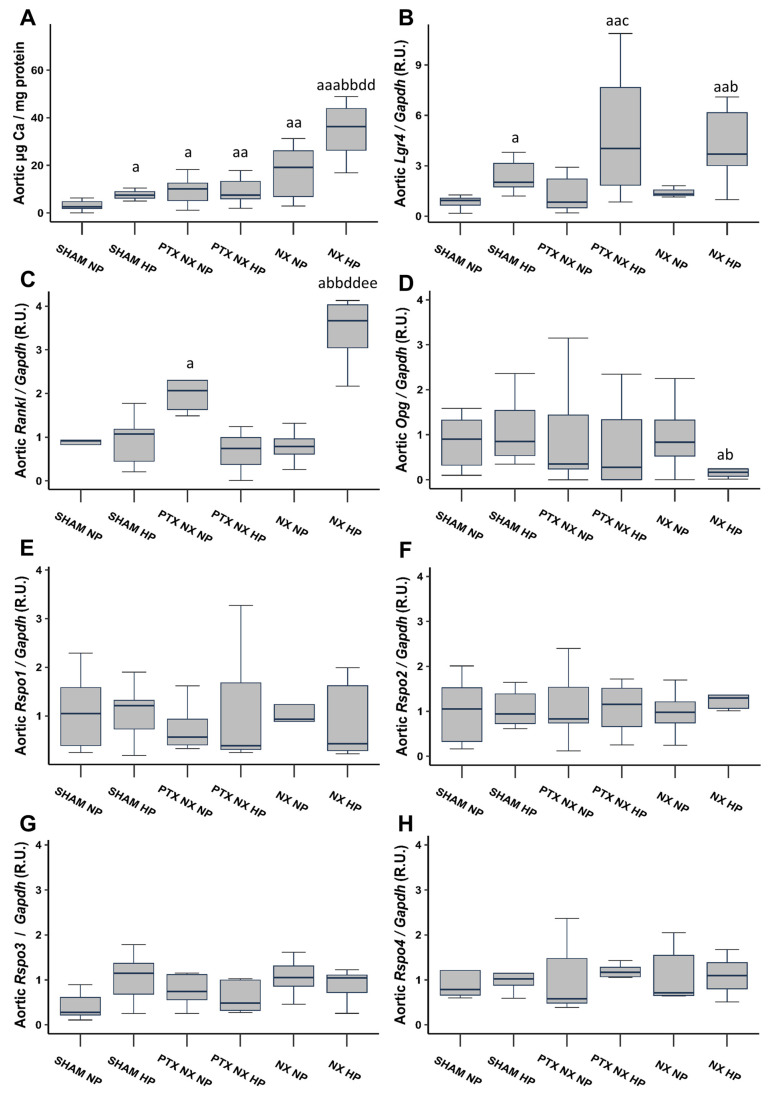
Aortic Ca content and gene expression from control (SHAM) and nephrectomized (NX) rats with and without parathyroidectomy, fed normal (NP) or high (HP) phosphorus diet for 18 weeks. Aortic Ca content was determined by o-cresolphtalein complexone method (**A**). Gene expression was evaluated by qRT-PCR of: *Lgr4* (**B**), *Rankl* (**C**), *Opg* (**D**), *Rspo1* (**E**), *Rspo2* (**F**), *Rspo3* (**G**) and *Rspo4* (**H**). The groups are SHAM NP (Sham-operated rats fed normal phosphorus diet), SHAM HP (Sham-operated rats fed high phosphorus diet), PTX NX NP (parathyroidectomized and nephrectomized rats fed normal phosphorus diet), PTX NX HP (parathyroidectomized and nephrectomized rats fed high phosphorus diet), NX NP (nephrectomized rats fed normal phosphorus diet), and NX HP (nephrectomized rats fed high phosphorus diet). Data are presented as median [interquartile range]. R.U., relative units. ^a^ *p* < 0.05, ^aa^ *p* < 0.01, ^aaa^ *p* < 0.001 versus SHAM NP; ^b^ *p* < 0.05, ^bb^ *p* < 0.01, versus SHAM HP; ^c^ *p* < 0.05 versus PTX NX NP; ^dd^ *p* < 0.01 versus PTX NX HP; ^ee^ *p* < 0.01 versus NX NP.

After 18 weeks, Sham rats fed an HP diet, and all nephrectomized (NX) groups exhibited a significant increase in the aortic Ca content compared to the Sham group fed an NP diet (Figure 1A). Notably, the increase was more pronounced in the NX group subjected to an HP diet (NX HP). The parathyroidectomy (PTX NX HP group) significantly reduced the markedly elevated aortic Ca content observed in the NX HP group.

A significant increase in systolic blood pressure (SBP) was observed in the PTX NX HP, NX NP, and NX HP groups compared to the Sham NP and Sham HP groups. This increase was particularly pronounced in the NX HP group. Conversely, diastolic blood pressure (DBP) showed a significant increase only in the NX NP and NX HP groups (Table 2).

#### 2.1.3. Aortic Gene Expression of Lgr4, Rankl, Opg, and Rspos

Aortic gene expression of *Lgr4* was significantly higher in all groups of rats fed an HP diet (Figure 1B). Aortic gene expression of *Rankl* significantly increased in the PTX NX NP and NX HP groups, with the greatest increase observed in the latter (Figure 1C). Furthermore, the NX HP group was the only group that exhibited a reduction in aortic *Opg* gene expression, although this reduction reached significance only when compared to the two Sham groups (Figure 1D). Finally, aortic gene expression of the Lgr4 ligands, *Rspos* (*Rspos1*, *2*, *3,* and *4*), did not show any significant change (Figure 1E–H).

#### 2.1.4. Serum Levels of LGR4, RANKL and OPG

Serum levels of OPG and LGR4 did not exhibit significant differences among the groups (Table 3). However, a notable increase in serum RANKL levels was observed in all groups of rats subjected to nephrectomy (NX groups). Consequently, the RANKL/OPG ratio was significantly higher in the PTX NX NP, NX NP, and NX HP groups, particularly in the latter (Table 3).

### 2.2. In Vitro Study

#### 2.2.1. VSMC Calcification and RANKL, OPG, LGR4, and RSPOs Expression

**Figure 2:**
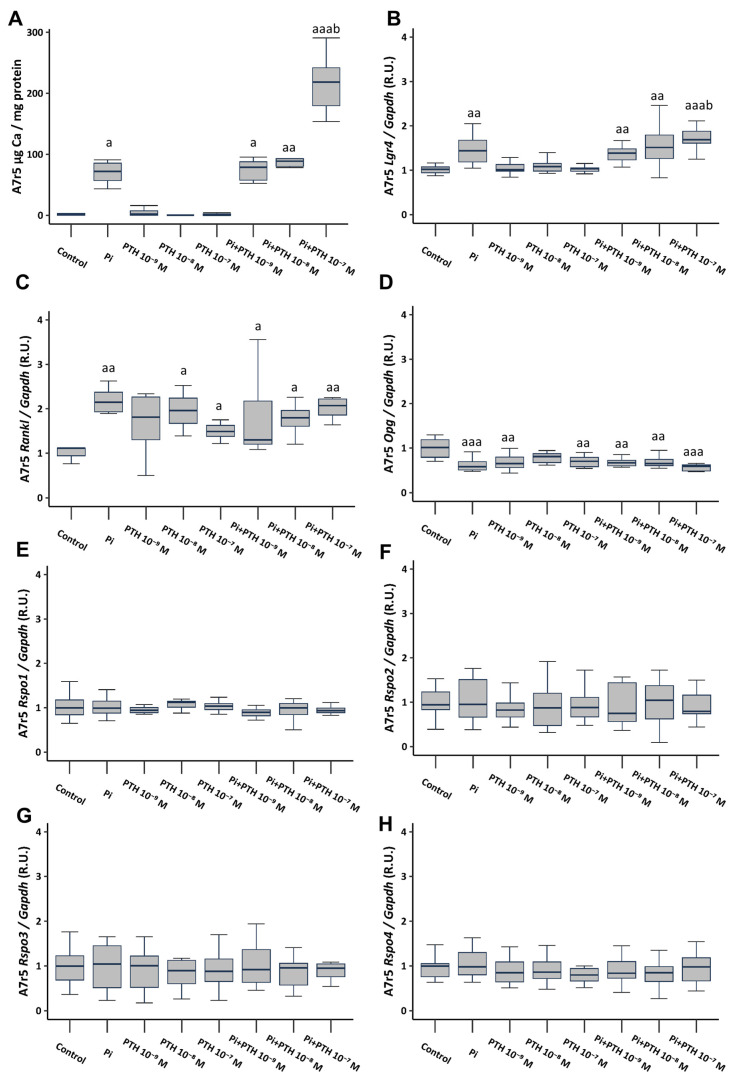
Effect of high phosphate (Pi) (3 mM) and different PTH concentrations (10^−9^, 10^−8^, and 10^−7^ M) on A7r5 vascular smooth muscle cells after 4 days of exposure. Ca content was determined by the o-cresolphtalein complexone method (**A**). Gene expression was evaluated by qRT-PCR for Lgr4 (**B**), Rankl (**C**), Opg (**D**), Rspo1 (**E**), Rspo2 (**F**), Rspo3 (**G**) and Rspo4 (**H**). Data are presented as median [interquartile range]. R.U., relative units. ^a^ *p* < 0.05, ^aa^ *p* < 0.01, ^aaa^ *p* < 0.001 versus control (non-calcifying medium, 1 mM phosphate); ^b^ *p* < 0.05 versus Pi.

The culture of VSMCs in calcifying medium (3 mM phosphate, referred to as Pi) resulted in a significant increase in Ca content, as well as a significant increase in the gene expression of Lgr4 and Rankl, and a significant decrease in Opg gene expression (Figure 2A–D). VSMCs cultured in a non-calcifying medium (1 mM phosphate) supplemented with different PTH concentrations (10^−9^, 10^−8^, and 10^−7^ M), did not result in changes in Ca content or Lgr4 expression (Figure 2A,B). However, it induced an increase in Rankl gene expression (Figure 2C) and a decrease in Opg expression (Figure 2D). The culture of VSMCs in calcifying medium (3 mM phosphate) supplemented with PTH concentrations (10^−9^, 10^−8^ and 10^−7^ M) showed no additional effect compared to phosphate alone, with the exception of the concentration of 10^−7^ M PTH, which triggered a significant increase in Ca content (81-fold increase compared to non-calcifying medium, and 2.5-fold increase compared to calcifying medium) (Figure 2A), and in Lgr4 gene expression (Figure 2B). None of the analyzed culture conditions induced changes in the gene expression of Rspo1, Rspo2, Rspo3, and Rspo4 (Figure 2E–H).

When analyzing the effect of calcifying medium (3 mM phosphate) on protein expression via Western Blot analysis, consistent findings emerged: heightened levels of LGR4 and RANKL accompanied by reduced OPG expression. For the protein studies, only a concentration of 10^−7^ M PTH was utilized due to its substantial influence on gene expression. The addition of 10^−7^ M PTH to non-calcifying medium (1 mM phosphate) induced an increase in RANKL levels, whereas its addition to calcifying medium (3 mM phosphate) induced the most significant upregulation of LGR4 and RANKL alongside a decrease in OPG expression. Additionally, consistent with previous observations, no changes were noted in RSPO levels (Figure S1).

#### 2.2.2. RANKL-LGR4 Calcification Pathway

**Figure 3:**
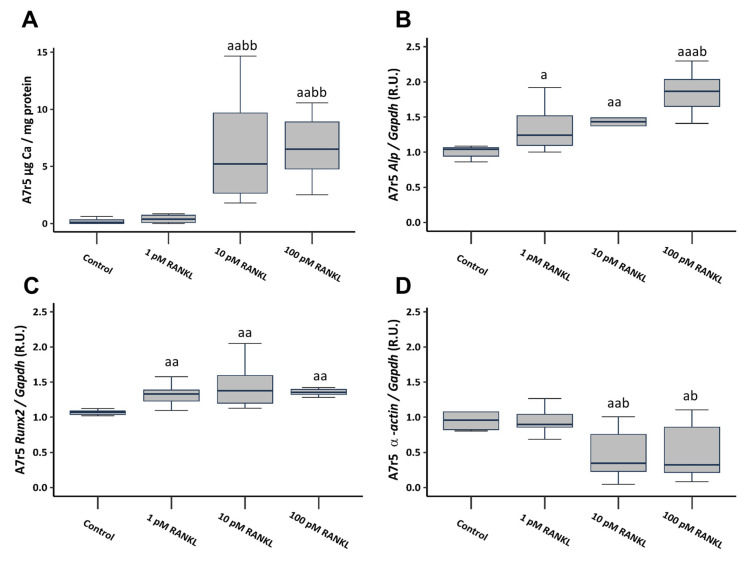
Effect of different soluble RANKL concentrations (1, 10, and 100 pM) on A7r5 vascular smooth muscle cells after 4 days of exposure in non-calcifying medium (1 mM phosphate). Ca content determined by o-cresolphtalein complexone method (**A**). Gene expression was evaluated by qRT-PCR for Alp (**B**), Runx2 (**C**), and α-actin (**D**). Data are presented as median [interquartile range]. R.U., relative units. ^a^ *p* < 0.05, ^aa^ *p* < 0.01, ^aaa^ *p* < 0.001 versus control (non-calcifying medium, 1 mM phosphate); ^b^ *p* < 0.05, ^bb^ *p* < 0.01, versus non-calcifying medium (1 mM phosphate) + 1 pM RANKL.

To investigate the involvement of RANKL in VSMCs calcification, soluble RANKL was added to a non-calcifying culture medium (1 mM phosphate) at concentrations of 1, 10, and 100 pM. The introduction of soluble RANKL to a non-calcifying medium resulted in a notable increase in Ca content, reaching significance starting from a concentration of 10 pM (Figure 3A). Additionally, it prompted heightened gene expression of osteogenic markers such as alkaline phosphatase (Alp) and RUNX family transcription factor 2 (Runx2), accompanied by a significant decrease in α-actin gene expression, starting from the 10 pM concentration (Figure 3B–D).

**Figure 4:**
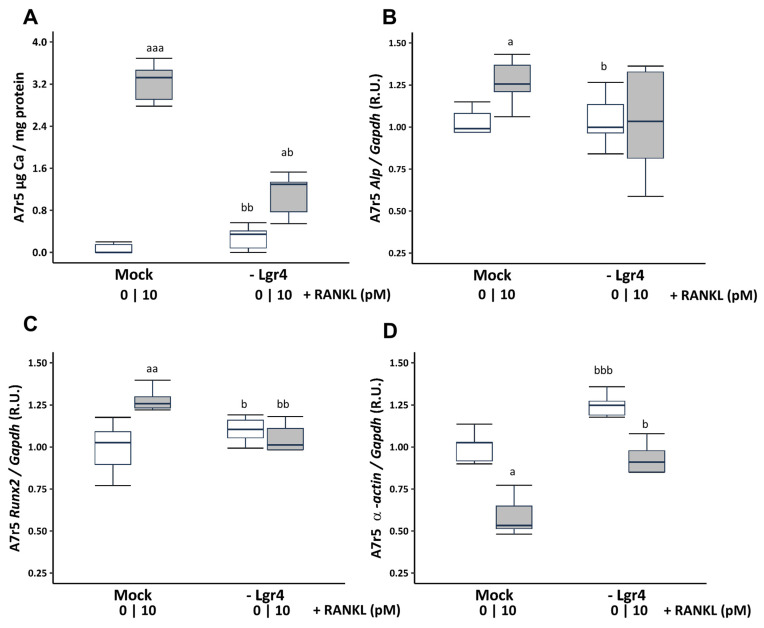
Effect of Lgr4 silencing on A7r5 vascular smooth muscle cells exposed to soluble RANKL (10 pM) in non-calcifying medium (1 mM phosphate). Ca content was determined by the o-cresolphtalein complexone method (**A**). Gene expression was evaluated by qRT-PCR for Alp (**B**), Runx2 (**C**), and α-actin (**D**). White and grey boxes represent the non-addition of RANKL and the addition of 10 pM of RANKL, respectively. Data are presented as median [interquartile range]. R.U., relative units. Mock, scramble transfection control of silencing. ^a^ *p* < 0.05, ^aa^ *p* < 0.01, ^aaa^ *p* < 0.001 versus Mock (0 pM RANKL); ^b^ *p* < 0.05, ^bb^ *p* < 0.01, ^bbb^ *p* < 0.001 versus Mock + 10 pM RANKL.

To ascertain whether these effects were mediated via RANKL binding to its receptor LGR4, Lgr4 was silenced in VSMCs cultured in a non-calcifying medium (1 mM phosphate). Receptor silencing resulted in a reduction of 73.81% ± 3.26 at 4 days compared to the scramble transfection (Mock). Lgr4 silencing prevented the increase in Ca content (Figure 4A) and the gene expression changes in Runx2 and α-actin, but not in Alp expression induced by 10 pM soluble RANKL (Figure 4B–D).

Increasing concentrations of soluble RANKL added to the calcifying culture medium (3 mM phosphate) resulted in a significantly greater increase in Ca content compared to phosphate alone, starting from a concentration of 10 pM RANKL (Figure S2A). The expression of the studied genes was already altered by the effect of the calcifying medium, with only a further increase in the expression of Runx2 observed with the addition of soluble RANKL starting from a concentration of 10 pM (Figure S2B). Silencing Lgr4 in VSMCs in calcifying medium (3 mM phosphate) partially prevented both the increase in Ca content induced by high phosphate and the upregulation of Runx2 induced by 10 pM soluble RANKL (Figure S2C,D).

### 2.3. Human Epigastric Arteries Study

No significant differences in sex and age were observed between patients without (Kauppila Index, KI = 0) or with vascular calcification (KI ≥ 1) (Table 4). The epigastric Ca content was significantly higher in the group with KI ≥ 1. Only the gene expression of RANKL was found to be significantly higher in the human epigastric arteries with vascular calcification (Kauppila Index, KI ≥ 1) (Table 4). The gene expression levels of LGR4, OPG, and RSPOs showed no significant differences.

## 3. Discussion

The present in vivo and in vitro study represents the first description of the influence of different LGR4 ligands, including RANKL and RSPOs, in the process of vascular calcification. The results suggest that among the analyzed ligands, only RANKL plays a significant role in LGR4-mediated calcification.

Vascular calcification, closely associated with decreased bone mass, is a common complication and a leading cause of mortality among the elderly, particularly in patients with chronic kidney disease (CKD) [21,22]. The RANK/RANKL/OPG system, along with the recently identified member LGR4, plays a crucial role in both bone and vascular mineralization. While its role in bone maintenance is well understood [23], the implications of this system in VSMC calcification remain poorly understood. In the vasculature, the increase in RANKL and the decrease in OPG have been demonstrated to promote calcification [10,11]. In fact, OPG knockout mice exhibit osteoporosis and severe arterial calcification [24]. Therefore, it was expected that Denosumab, a human monoclonal antibody against RANKL used for osteoporosis treatment, would be capable of preventing or at least delaying the progression of vascular calcification. Indeed, studies have shown that Denosumab reduces vascular Ca deposition in mice with glucocorticoid-induced osteoporosis, further emphasizing the link between bone and the vascular system [25,26]. However, the FREEDOM study, which involved osteoporotic patients, revealed that the frequency of aortic calcification progression and adverse cardiovascular events was similar between women in the placebo and Denosumab-treated groups. Nevertheless, the treatment did improve bone mineral density and reduced fracture risk [27]. These findings underscore the necessity for further research to elucidate the mechanisms regulating this pathway in both bone and the vasculature to prevent strategies aimed at protecting bone from inadvertently exacerbating vascular calcification.

The identification of a novel receptor for RANKL, LGR4, marked a significant advancement in understanding bone formation regulation [8]. LGR4 plays a pivotal role in enhancing bone formation by promoting osteoblast maturation and mineralization [8,19,28]. This receptor promotes the activation of the Wnt/β-catenin pathway and regulates the expression of osteogenic differentiation markers such as RUNX2 and ALP [19]. Moreover, the extracellular domain of LGR4 binds RANKL, competing with RANK, thereby inhibiting osteoclast differentiation and reducing osteoclastic bone resorption. Studies involving LGR4 (−/−) mice have demonstrated a delay in osteoblast differentiation and mineralization during embryonic bone development. Furthermore, postnatal bone remodeling is markedly compromised in LGR4 (−/−) mice due to reduced osteoid formation and increased osteoclast activity, leading to decreased bone formation rates and diminished bone mineral density [28]. Additionally, in humans, a nonsense mutation in LGR4 has been strongly correlated with low bone mineral density and osteoporotic fractures [9]. As elucidated, while LGR4 plays a crucial role in bone formation, its dysregulation may also potentially aggravate vascular calcification.

Previous studies from our group have delineated the involvement of LGR4 in the vascular calcification process [12]. The aortic gene expression of LGR4 significantly increased in uremic rats in response to elevated phosphate and PTH levels, predominantly localizing within calcified aortic regions. Crucially, deletion of the *Lgr4* gene in VSMCs impeded phosphate/PTH-induced calcification.

In the current investigation, nephrectomized (NX) rats on a high phosphorus diet exhibited elevated aortic Ca content along with increased expression levels of *Rankl* and *Lgr4* and reduced expression of *Opg*. Furthermore, PTH has been identified as a major regulator of this system, inducing upregulation of *Rankl* expression and downregulation of *Opg* [5,6,7]. Additionally, NX rats fed a high phosphorus diet also displayed elevated serum PTH levels concomitant with augmented aortic expression of *Rankl* and *Lgr4* and diminished expression of *Opg*. The implantation of pellets in parathyroidectomized rats (PTX) maintained normal serum PTH (1–34) levels, comparable to those of the Sham-operated group, but only partially mitigated the upregulation of *Rankl*, and the downregulation of *Opg*. Also, the parathyroidectomy significantly attenuated the notably elevated aortic Ca content observed in the NX HP group. In fact, previous studies have demonstrated a link between elevated PTH levels and increased vascular calcification through mechanisms involving osteogenic differentiation and mineralization processes within VSMCs [16,29,30]. These findings are further supported by in vitro studies, which showed that high phosphate levels promote VSMCs differentiation (evidenced by upregulation of the osteogenic markers *Osterix* and *Runx2*, and downregulation of *α-actin*, as shown in Tables S1 and S2) and calcification. This process is accompanied by upregulation of *Lgr4* and *Rankl* and downregulation of *Opg*, with elevated PTH levels intensifying these effects.

A review of the literature reveals that uremic rats require approximately nine times more PTH to maintain normal serum Ca levels compared to controls [31]. However, despite elevated PTH levels, normalization of Ca levels is not achieved when phosphate levels are elevated (due to dietary intake) [32]. Furthermore, high phosphorus diets induced an increase in serum calcitriol levels in all groups, except for the PTX rats, underscoring the critical role of PTH in renal calcitriol production [33]. This suggests that an elevation in PTH is essential for the induction of calcitriol production.

Another relevant finding of the study is that the PTX NX HP group exhibited the highest serum phosphate values, likely due to the necessity of elevating PTH to exert its phosphaturic actions and facilitate phosphate excretion in the urine [34]. Surprisingly, this higher serum phosphate level did not stimulate an increase in FGF23. Additionally, it is well known that an excess of phosphate induces a decrease in serum Ca (as observed in this group of animals), and this Ca deficit reduces circulating levels of FGF23 [35], which may be happening in the PTX NX HP group, thus compensating for FGF23 levels. These data suggest that not only phosphate and Ca levels regulate FGF23 but also PTH may exert some control [36].

On the other hand, rats with higher aortic Ca content were not necessarily those with the highest serum phosphate levels or the highest Ca × P product; rather, the most calcified rats were those exhibiting an increase in serum phosphate accompanied by a significant elevation of PTH. This again underscores the pivotal role of PTH in vascular calcification. These findings highlight the importance of controlling both serum phosphate and PTH levels within optimal ranges in CKD patients to maintain healthy vasculature.

Systolic blood pressure (SBP) pressure increased in parallel with the aortic Ca content, particularly in the most calcified animals (NX HP), suggesting increased arterial stiffness. Conversely, parathyroidectomy (PTX) partially prevented the increase in SBP. Although elevated levels of PTH have been associated with hypertension [37,38], paradoxically, PTH also exhibits vasodilatory and natriuretic effects. Therefore, it appears that the elevation of PTH seeks to counterbalance the rise in blood pressure in these patients [37]. The results of the present study demonstrated that elevated PTH levels significantly increased aortic Ca content (a finding corroborated by in vitro studies) and led to greater arterial stiffness and higher SBP.

The role of RANKL and OPG as serum biomarkers has been extensively reported, with the RANKL/OPG ratio being crucial for assessing bone remodeling and bone mass [39]. However, there is no consensus on its relationship to vascular calcification. Different studies with CKD patients found an association between vascular calcification and high OPG serum levels [40,41]. In contrast, another study involving patients with ischemic coronary disease found a negative correlation between circulating OPG levels and total coronary artery calcification, no correlation with serum RANKL concentration, and a positive correlation between RANKL/OPG ratio and total coronary artery calcification [42]. Additionally, recent research has shown positive associations between serum RANKL levels and coronary artery calcification in a cohort of stable ambulatory patients without known coronary artery disease who underwent coronary computed tomography [43]. These divergent results might be attributed to differences in disease etiology, the disease stage at the time of biomarker measurement, and the indeterminate origins of these biomarkers—whether they originate from bone undergoing mineralization, vessels undergoing calcification, or vessels being protected from calcification.

In the rats of the present study, RANKL serum levels were elevated in all nephrectomized groups, although there are no significant differences in OPG serum levels among the different groups. However, the NX HP group, which had the highest aortic Ca content, presented the highest RANKL/OPG ratio. This finding supports the hypothesis that the RANKL/OPG ratio is a possible serum biomarker of vascular calcification.

In human epigastric arteries from renal transplant recipients, the gene expression of *LGR4*, *RANKL*, *OPG*, and *RSPOs* was analyzed. Vascular calcification was assessed using the semi-quantitative Kauppila Index (KI) based on lateral abdominal X-ray images. While interpreting these results, caution is advised due to the inherent challenges in extrapolating findings from animal models directly to human conditions. Notably, in arteries from individuals with a KI ≥ 1, indicating the presence of vascular calcification, only the gene expression of *RANKL* was significantly elevated, highlighting its pivotal role in the development of vascular calcification. Unfortunately, the unavailability of serum samples from these patients restricted our ability to measure circulating levels of RANKL and OPG, presenting a major limitation of this study.

A recent study has suggested that LGR4 can be detected in serum [44] where it is hypothesized to sequester RANKL preventing its binding to RANK. However, the biological relevance of this mechanism has not been determined, and the authors suggest further studies to investigate the possible role of LGR4 as a serum biomarker for bone loss or vascular calcification. In our animal model, LGR4 was measured in the serum samples from different groups of rats. However, no significant differences were found, suggesting that LGR4 may not serve as a serum biomarker for vascular calcification.

LGR4 exhibits multiple ligands aside from RANKL. Recently, it has been elucidated that one of these ligands is Nidogen-2, a basement membrane protein that engages LGR4 to activate the Gαq-PKCα-AMPKα1 signaling pathway, subsequently inhibiting the vascular calcification [45]. Additionally, LGR4 serves as a receptor for the R-spondins (RSPOs) [19,20], which play significant actions in bone and form a complex with well-known Wnt modulators Frizzled/Lrp [46]. All RSPOs have demonstrated the ability to enhance Wnt/β-catenin signaling by increasing the phosphorylation of Wnt coreceptors LRP5/6 [47]. Given the recent interest in the role of LGR4 in vascular calcification and the close interconnection between bone and vasculature, it seemed intriguing to explore the role of RSPO ligands in this process. However, in all in vivo and in vitro models utilized in the present study, none of the RSPOs were found to modulate vascular calcification. The only ligand studied that exhibited modulation was RANKL, suggesting that RANKL may play a more relevant role in inducing VSMC calcification.

It is well established that the interaction between RANKL and RANK initiates a signaling and gene expression cascade, activating the transcription factor NF-κB, which promotes VSMC calcification [48]. However, there is limited information regarding the interaction between RANKL and LGR4 in vascular calcification. In the present study, we investigated whether the increase in RANKL induces vascular calcification through LGR4. RANKL increased Ca content in VSMCs under both calcifying and non-calcifying conditions and promoted osteogenic differentiation of VSMCs by reducing the expression of the contractile phenotype marker *α-actin* and increasing the expression of the osteogenic factors *Runx2* and *Alp*. Partial inhibition of these effects induced by RANKL was observed upon *Lgr4* silencing, indicating that the binding of RANKL to LGR4 would promote osteogenic differentiation and calcification of VSMCs. This finding introduces a novel perspective on the role of RANKL in vascular calcification, suggesting that elevated phosphate levels may enhance RANKL secretion, which then acts in an autocrine/paracrine manner on LGR4 in VSMCs, alongside RANK, to promote calcification. This could elucidate a novel mechanism by which RANKL participates in the process of vascular calcification independently of the RANKL-RANK binding. Therefore, RANKL may exert its pro-calcifying actions in VSMCs through both RANK and LGR4 binding. However, an important limitation of our study is that while we demonstrate the interaction between RANKL and LGR4 in VSMCs, the specific signaling pathways activated by this interaction, which ultimately disrupts *Runx2*, *Alp*, and *α-actin* gene expression leading to mineralization, remain unclear. Identifying these pathways could further delineate the pro-calcifying mechanisms of RANKL in vascular contexts.

In summary, although RSPOs have been described as having an important role in bone formation, their contribution to vascular calcification did not appear significant based on the in vivo and in vitro models presented in the current study. However, the interaction between RANKL and LGR4, which enhances the osteogenic differentiation of VSMCs, represents a novel mechanism of action. Consequently, targeting LGR4 could emerge as a promising therapeutic strategy aimed at preventing or mitigating vascular calcification.

## 4. Materials and Methods

### 4.1. Rat Experimental Study

#### 4.1.1. Animal Model

The experimental procedures were conducted in accordance with the guidelines and protocols approved by the Laboratory Animal Ethics Committee of Oviedo University (PROAE14/2021).

Male Wistar rats aged 4 months and weighing between 350–400 g were used. Initially, one group of rats underwent microsurgical parathyroidectomy (PTX), while a Sham operation mimicking PTX was performed on the remaining rats. In the PTX group, only rats with intact PTH [PTH (1–84)] serum levels below 50 pg/mL were considered to have undergone successful PTX, resulting in an 83.3% success rate. Subcutaneous pellets (supplied by Innovative Research of America, Sarasota, FL, USA) were implanted in all PTX animals to facilitate continuous infusion of PTH (1–34) at a dosage of 5 µg/kg/day. The objective was to attain normal serum levels of PTH (1–34). In the PTX Sham-operated rats, subcutaneous pellets containing vehicle (provided by Sigma-Aldrich, St. Louis, MO, USA) were implanted. Ten days later, chronic renal failure (CRF) was induced in the PTX group and in a group of PTX Sham-operated rats by partial nephrectomy (NX), involving the removal of the right kidney and three-quarters of the left kidney [49].

The NX animals, whether subjected to parathyroidectomy (PTX) or not, were divided based on their dietary regimen: normal phosphorus (NP) diet containing 0.6% phosphorus (P) and 0.6% calcium (Ca), or a high phosphorus (HP) diet containing 0.9% P and 0.6% Ca, both sourced from Panlab, Barcelona, Spain. The rats were housed in wire cages with unrestricted access to food and water. Thus, the study consisted of 6 groups of animals: SHAM NP, SHAM HP, PTX NX NP, PTX NX HP, NX NP, NX HP.

After 18 weeks, the arterial blood pressure was measured, and the rats were placed in metabolic cages for a 24 h period to facilitate urine collection before they were euthanized by exsanguination under isoflurane anesthesia. Subsequently, serum samples were obtained for further analyses. The aortas were excised, rinsed twice with a saline solution, and divided into two segments. These segments were used to determine Ca content and RNA extraction.

#### 4.1.2. Biochemical Markers

Serum creatinine, phosphate, Ca, and urinary creatinine were measured using a multichannel autoanalyzer (Hitachi 717: Boehringer Mannheim, Berlin, Germany). Serum calcitriol was measured by RIA (Immunodiagnostic Systems, Tyne & Wear, UK). Serum PTH (1–84), PTH (1–34), FGF23, RANKL, OPG and LGR4 were measured by an enzyme-linked immunosorbent assay (ELISA); (PTH (1–84): Quidel, San Diego, CA, USA; PTH (1–34): MyBioSource, San Diego, CA, USA; FGF23: Kainos Laboratories, Tokyo, Japan; RANKL and OPG: R&D Systems, Minneapolis, MN, USA; LGR4: MyBioSource).

#### 4.1.3. Arterial Blood Pressure Measurement

Systolic (SBP) and diastolic blood pressure (DBP) were assessed during the last week of the study using the LE 5002 Blood Pressure Meter, an automated, non-invasive tail-cuff method (Panlab). To minimize stress induced by the procedure, the animals underwent a four-day acclimation period with the instrument before the final measurements. Each measurement consisted of a minimum of 10 repetitive readings per rat.

### 4.2. In Vitro Experimental Study

#### 4.2.1. A7r5 Cell Culture

The embryonic rat cell line A7r5 is commonly employed for studying cell calcification in the tunica media of arteries in vitro [50]. Rat vascular smooth muscle cells (A7r5, ATCC, Manassas, VA, USA) were seeded at a density of 10,000 cells/cm^2^ in six-well plates (Corning Costar, Corning, NY, USA) and cultured in Dulbecco’s Modified Eagle Medium (DMEM) supplemented with 10% fetal bovine serum (FBS) and 1% penicillin/streptomycin (Lonza, Bornem, Belgium) until they reached subconfluence. Subsequently, the growth medium was replaced with either DMEM containing 1 mM phosphate as the control medium or DMEM supplemented to achieve a final concentration of 3 mM phosphate (referred to as the calcifying medium [51,52]), both supplemented with 1% FBS. The cells were also exposed to three different concentrations of PTH (1–34) (10^−9^, 10^−8^, and 10^−7^ M) for 4 days [16], with the medium refreshed every 48 h. At the end of the experiment, cells were harvested for the quantification of Ca deposition and for the collection of total RNA and protein.

In additional experiments, A7r5 cells were exposed to increasing concentrations of soluble RANKL recombinant protein (Gibco, Waltham, MA, USA) (1, 10, and 100 pM) [10] for the same 4-day period in both control medium (1 mM phosphate) and calcifying medium (3 mM phosphate). Cells were then collected to measure Ca deposition and to obtain total RNA.

At least three replicates of each experiment were performed in triplicate.

#### 4.2.2. Small Interfering RNA (siRNA) for LGR4 Gene

A7r5 cells were seeded at a density of 10,000 cells/cm^2^ in six-well plates. After 24 h, when they reached 60–70% confluence, the cells were transfected overnight with a Smart Pool (Table S3). siRNA targeting the LGR4 gene (Thermo Fisher Scientific, Waltham, MA, USA) using the DharmaFECT transfection reagent (Horizon Discovery, Cambridge, UK) according to the manufacturer’s instructions. A transfection control with a scramble sequence (Mock) was employed, using the same concentrations and exposure times as a negative control. Subsequently, A7r5 cells were cultured for 4 days in a control medium (1 mM phosphate) and calcifying medium (3 mM phosphate) with or without 10 pM RANKL. The levels of Ca deposition and the expression of phenotypic differentiation and osteogenic genes (*α-actin*, alkaline phosphatase -*Alp*- and RUNX family transcription factor 2 -*Runx2*-) were then assessed. At least three replicates of each experiment were performed in triplicate.

### 4.3. Human Epigastric Arteries Study

#### 4.3.1. Patients and Samples

A total of 41 fragments of epigastric arteries obtained from kidney transplant recipients were included in this study. A section of the epigastric artery was used for RNA extraction. All patients provided their informed consent to participate. The study was conducted according to the Declaration of Helsinki and approved by the Research Ethics Committee of the Principality of Asturias (Code 42/2011 approved on 28 April 2011).

#### 4.3.2. Patients and Samples Assessment of Vascular Calcification

Vascular calcification in the aortas of kidney transplant recipients was evaluated using the semi-quantitative Kauppila Index (KI) based on a lateral radiograph of the lumbosacral spine, assessing the presence of calcification in the anterior and posterior walls of the aorta at the level of lumbar vertebrae L1 to L4 [53]. A single radiologist, blinded to the patient’s data, assessed all X-ray images with an intra-observer coefficient of variation below 2%. A KI score greater than 0 was indicative of the presence of vascular calcification.

The Ca content in an artery section was also determined using the o-cresolphthalein complexone method.

### 4.4. Technical Procedures

#### 4.4.1. Ca Content Quantification

A 5-mm segment of the abdominal aorta, a fragment of human epigastric artery or A7r5 cells were homogenized in 0.6 N NaCl and gently agitated at 4 °C for 24 h. After centrifugation of the samples (9300× *g*), Ca content in the supernatant was determined using the o-cresolphthalein complexone method (Sigma-Aldrich). The pellet was resuspended in lysis buffer (containing 125 mM Tris and 2% sodium dodecyl sulfate (SDS) at pH 6.8) for protein extraction, and protein levels were quantified using the DC protein assay (Bio-Rad Laboratories, Hercules, CA, USA). The Ca content was normalized to total cell protein and expressed as µg Ca/mg protein.

#### 4.4.2. Total RNA Isolation, cDNA Synthesis, and Quantitative RT-PCR

A fragment of the abdominal aorta from the rats and one of the human epigastric arteries were homogenized using an ultraturrax (IKA-Werke, Staufen, Germany) in TRI Reagent (Sigma Aldrich), following the manufacturer’s guidelines. A7r5 cells were also collected in TRI Reagent. The total RNA concentration and purity were assessed using ultraviolet-visible spectrophotometry (NanoDrop Technologies, Wilmington, DE, USA) by measuring absorbance at 260 and 280 nm. Reverse transcription was conducted using a High-Capacity cDNA Reverse Transcription Kit (Applied Biosystems, Waltham, MA, USA) as per the manufacturer’s instructions. Gene expression was quantified through quantitative reverse transcription polymerase chain reaction (qRT-PCR) using a QuantStudio^TM^ 3 Real-Time PCR (Applied Biosystems). TaqMan real-time polymerase chain reaction (PCR) amplification was performed with gene-specific primers (gene expression assays from Applied Biosystems) targeting *Rspo1*, *Rspo2*, *Rspo3*, *Rspo4*, *Rankl*, *Opg*, *Lgr4*, *α-actin*, *Alp*, and *Runx2* (Table S4). Glyceraldehyde-3-phosphate dehydrogenase (*Gapdh*) was employed as a housekeeping gene. Relative quantitative analysis of target genes was conducted by comparing threshold cycles using the ΔΔCt method.

#### 4.4.3. Western Blot

Total protein extracts from A7r5 cells were collected, quantified using the DC assay (Bio-Rad), and 20 µg of protein were separated via SDS–polyacrylamide gel electrophoresis (10%), followed by transfer onto polyvinylidene difluoride membranes (Hybond; Amersham, Amersham, UK). Western blot analyses were conducted following the manufacturer’s protocols with specific antibodies: RSPO1 (1:1000; Thermo Fisher), RSPO2 (1:500; LifeSpan BioSciences, Lynnwood, WA, USA), RSPO3 (1:500; Thermo Fisher), RSPO4 (1:1000; Abcam, Cambridge, UK), RANKL (1:500; Santa Cruz Biotechnology, Dallas, TX, USA), OPG (1:500; Santa Cruz Biotechnology), LGR4 (1:500; Santa Cruz Biotechnology), and GAPDH (dilution 1:3000; Santa Cruz Biotechnology). Secondary antibody binding was detected using the ECL Western Blotting Detection Kit (Amersham), and the ChemiDoc Gel Imaging System Model XRS (Bio-Rad) was employed for visualization and quantification with Quantity One 1-D analysis software version 4 (Bio-Rad).

### 4.5. Statistical Analysis

Due to the non-normal distribution of the variables, data in figures and tables are presented as median and interquartile range (IQR). Groups were compared using the non-parametric Kruskal–Wallis test with the post-hoc analysis Dunn’s Multiple Comparison test. All analyses were performed using R software 4.3.1.

### 4.6. Abbreviations

A list of all the abbreviations of the article has been detailed in Table S5.

## Figures and Tables

**Table 1:** Biochemical and renal function parameters in the different groups of rats. Creatinine clearance, serum phosphate, PTH (1–84), PTH (1–34), calcium, FGF23, and calcitriol. Data are represented as median [interquartile range (IQR)]. n, number of rats. ^a^ *p* < 0.05, ^aa^ *p* < 0.01, ^aaa^ *p* < 0.001 versus SHAM NP; ^bb^ *p* < 0.01, ^bbb^ *p* < 0.001 versus SHAM HP; ^c^ *p* < 0.05, ^cc^ *p* < 0.01, ^ccc^ *p* < 0.001 versus PTX NX NP; ^d^ *p* < 0.05, ^dd^ *p* < 0.01, ^ddd^ *p* < 0.001 versus PTX NX HP; ^ee^ *p* < 0.01 versus NX NP.

	SHAM NP	SHAM HP	PTX NX NP	PTX NX HP	NX NP	NX HP
n	9	11	8	13	7	8
Creatinine clearance (mL/min) (median [IQR])	2.99 [2.79, 3.30]	2.77 [2.23, 3.05]	1.01 [0.68, 1.40] ^aaa^	1.12 [0.69, 1.29] ^aaabbb^	0.97 [0.73, 1.20] ^aaa^	1.22 [1.01, 1.31] ^aaabb^
Serum Phosphate (mg/dL) (median [IQR])	3.94 [3.57, 4.06]	4.31 [4.06, 4.53]	5.70 [4.69, 6.41] ^aa^	9.27 [9.11, 9.92] ^aaabbbc^	3.88 [3.50, 4.73] ^ddd^	4.82 [4.46, 5.05] ^ad^
Serum PTH 1–84 (pg/mL) (median [IQR])	232.65 [183.70, 307.20]	410.15 [378.40, 613.08] ^a^	Undetectable	Undetectable	699.19 [603.44, 948.62] ^accc^	1195.50 [942.50, 1916.56] ^aaddd^
Serum PTH 1–34 (pg/mL) (median [IQR])	16.14 [13.19, 29.55]	25.21 [14.20, 33.00]	39.97 [27.82, 73.56]	5.09 [5.09, 34.62]	42.54 [32.00, 243.40]	504.00 [504.00, 608.20] ^aaabbddd^
Serum Calcium (mg/dL) (median [IQR])	10.23 [10.03, 10.31]	10.15 [9.98, 10.27]	9.72 [9.55, 9.92]	6.50 [6.18, 7.30] ^aaabbbc^	10.95 [10.61, 11.25] ^cc^	10.21 [10.13, 10.43] ^ddd^
Serum FGF23 (pg/mL) (median [IQR])	52.82 [41.18, 72.27]	67.68 [40.95, 77.80]	246.27 [143.00, 343.32] ^a^	94.82 [56.73, 189.82]	206.18 [133.59, 262.64] ^aa^	199.91 [182.55, 407.59] ^aabb^
Serum Calcitriol (pg/mL) (median [IQR])	5.00 [5.00, 5.15]	14.90 [12.65, 18.05] ^aaa^	5.00 [5.00, 5.00]	5.00 [5.00, 5.00] ^bbb^	5.00 [5.00, 5.00]	10.26 [8.08, 12.22] ^addee^

**Table 2:** Arterial blood pressure. SBP: Systolic blood pressure. DBP: Diastolic blood pressure. Data represent median [interquartile range (IQR)]. n, number of rats. ^a^ *p* < 0.05, ^aa^ *p* < 0.01, ^aaa^ *p* < 0.001 versus SHAM NP; ^b^ *p* < 0.05, ^bb^ *p* < 0.01 versus SHAM HP; ^c^ *p* < 0.05, versus PTX NX NP; ^d^ *p* < 0.05, versus PTX NX HP.

	SHAM NP	SHAM HP	PTX NX NP	PTX NX HP	NX NP	NX HP
n	9	11	8	13	7	8
SBP (Hg mm) (median [IQR])	119.00 [116.33, 126.90]	123.67 [117.58, 126.39]	124.96 [122.70, 129.68]	132.50 [125.25, 140.00] ^ab^	132.67 [131.73, 144.38] ^aa^	142.50 [134.27, 151.74] ^aaabbd^
DBP (Hg mm) (median [IQR])	83.00 [78.00, 97.68]	92.71 [88.65, 97.36]	87.68 [76.00, 96.50]	96.75 [82.25, 108.17]	103.53 [99.85, 108.17] ^ac^	108.00 [101.33, 113.67] ^a^

**Table 3:** Serum levels of RANKL, OPG, LGR4, and RANKL/OPG ratio. Data are represented as median [interquartile range (IQR)]. n, number of rats. ^a^ *p* < 0.05, ^aa^ *p* < 0.01 versus SHAM NP; ^b^ *p* < 0.05, ^bb^ *p* < 0.01 versus SHAM HP.

	SHAM NP	SHAM HP	PTX NX NP	PTX NX HP	NX NP	NX HP
n	9	11	8	13	7	8
RANKL (pg/mL) (median [IQR])	18.64 [14.13, 24.79]	25.57 [17.58, 35.09]	64.24 [37.24, 87.16] ^aa^	65.43 [31.92, 75.57] ^ab^	53.76 [53.34, 69.71] ^aa^	64.59 [45.13, 79.83] ^aab^
OPG (pg/mL) (median [IQR])	2568.31 [2093.14, 3684.58]	3024.64 [2405.98, 4888.46]	3394.41 [2653.62, 4045.47]	3438.16 [2478.00, 4160.14]	3032.46 [2860.33, 3329.02]	2815.49 [2183.17, 3021.41]
RANKL/OPG (%)	0.73 [0.50, 0.96]	0.55 [0.36, 1.03]	2.23 [1.32, 2.88] ^a^	1.51 [0.69, 2.63]	1.98 [1.66, 2.42] ^a^	2.95 [1.43, 3.39] ^aabb^
LGR4 (ng/mL) (median [IQR])	3.23 [2.43, 3.75]	2.81 [1.92, 3.10]	3.26 [1.91, 4.94]	4.42 [3.41, 5.49]	3.09 [2.75, 4.20]	3.17 [2.74, 4.40]

**Table 4:** Human epigastric arteries study results. Relevant demographic parameters from 41 kidney transplant recipients in whom epigastric arteries were studied. Ca content and *LGR4*, *OPG*, *RANKL*, *RSPO1*, *RSPO2*, *RSPO3*, and *RSPO4* gene expression in the epigastric artery between patients without (KI = 0) or with vascular calcification (KI ≥ 1). KI: Kauppila Index. Data represent median [interquartile range (IQR)]. n, number of samples. R.U., relative units. * *p* < 0.05, ** *p* < 0.01 versus KI = 0.

	KI = 0	KI ≥ 1	*p*
n	10	31	
Sex = Female (%)	5 (50.0)	13 (41.9)	0.936
Age (years) (median [IQR])	56.50 [52.25, 59.00]	59.00 [56.50, 63.00]	0.166
Epigastric artery Ca content (µg Ca/mg protein) (median [IQR])	14.35 [10.48, 19.56]	48.84 [17.95, 376.83]	0.004 **
*LGR4/GAPDH* (R.U.) (median [IQR])	0.80 [0.40, 1.07]	0.59 [0.33, 1.29]	0.345
*OPG/GAPDH* (R.U.) (median [IQR])	0.79 [0.17, 1.16]	0.38 [0.24, 0.91]	0.851
*RANKL/GAPDH* (R.U.) (median [IQR])	1.17 [0.60, 1.32]	1.78 [1.02, 2.82]	0.035 *
*RSPO1/GAPDH* (R.U.) (median [IQR])	0.43 [0.15, 1.99]	0.29 [0.15, 0.54]	0.306
*RSPO2/GAPDH* (R.U.) (median [IQR])	0.66 [0.15, 1.45]	0.57 [0.23, 2.04]	0.935
*RSPO3/GAPDH* (R.U.) (median [IQR])	0.63 [0.22, 1.41]	0.55 [0.23, 0.82]	0.642
*RSPO4/GAPDH* (R.U.) (median [IQR])	0.62 [0.56, 1.28]	1.32 [0.60, 2.44]	0.130

## Data Availability

The data presented in this study are available on request from the corresponding authors.

## Supplementary Materials

The following supporting information can be downloaded at: https://www.mdpi.com/article/10.3390/ijms25115735/s1.
