# Supplementary material for: RANKL, but Not R-Spondins, Is Involved in Vascular Smooth Muscle Cell Calcification through LGR4 Interaction
Source: Int J Mol Sci. 2024 May 24;25(11):5735. doi: 10.3390/ijms25115735 (PMC11172097; doi:10.3390/ijms25115735)
Supplement: Supplementary file 1 [file ijms-25-05735-s001.zip › ijms-3002107-supplementary.pdf]

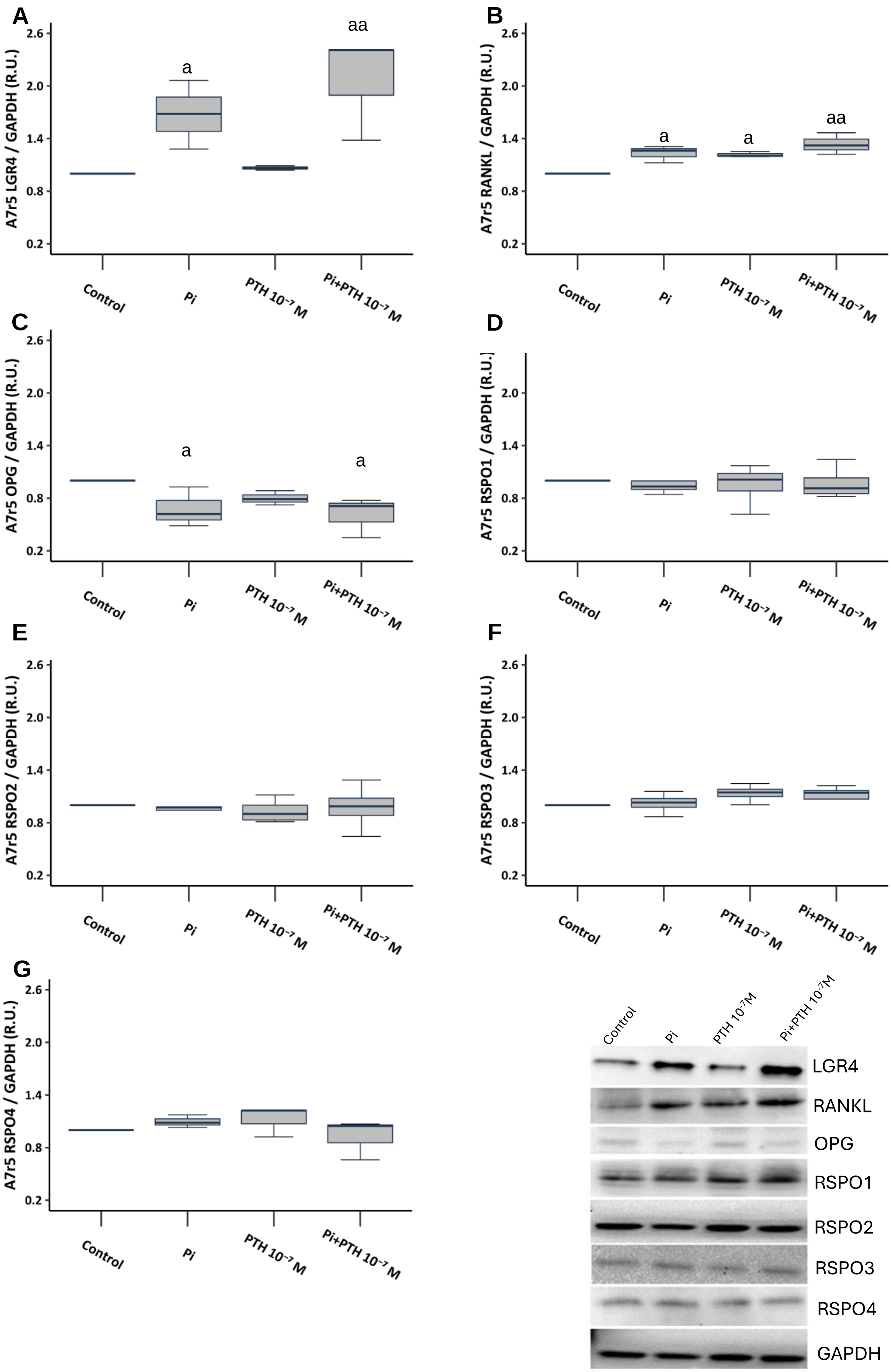

Figure S1: Effect of high phosphate (3 mM, Pi) and high PTH (10^{-7} M) addition on A7r5 vascular smooth muscle cells after 4 days of exposure. Protein levels determined by Western Blot of: LGR4 (**A**), RANKL (**B**), OPG (**C**), RSPO1 (**D**), RSPO2 (**E**), RSPO3 (**F**) and RSPO4 (**G**). Image representative of LGR4, RANKL, OPG, RSPOS1-4 and GAPDH (constitutive) protein levels evaluated by western blot (**H**). Data are presented as median [interquartile range]. R.U., relative units. $^{a}p < 0.05$, $^{aa}p < 0.01$ versus control (non-calcifying medium, 1 mM phosphate).

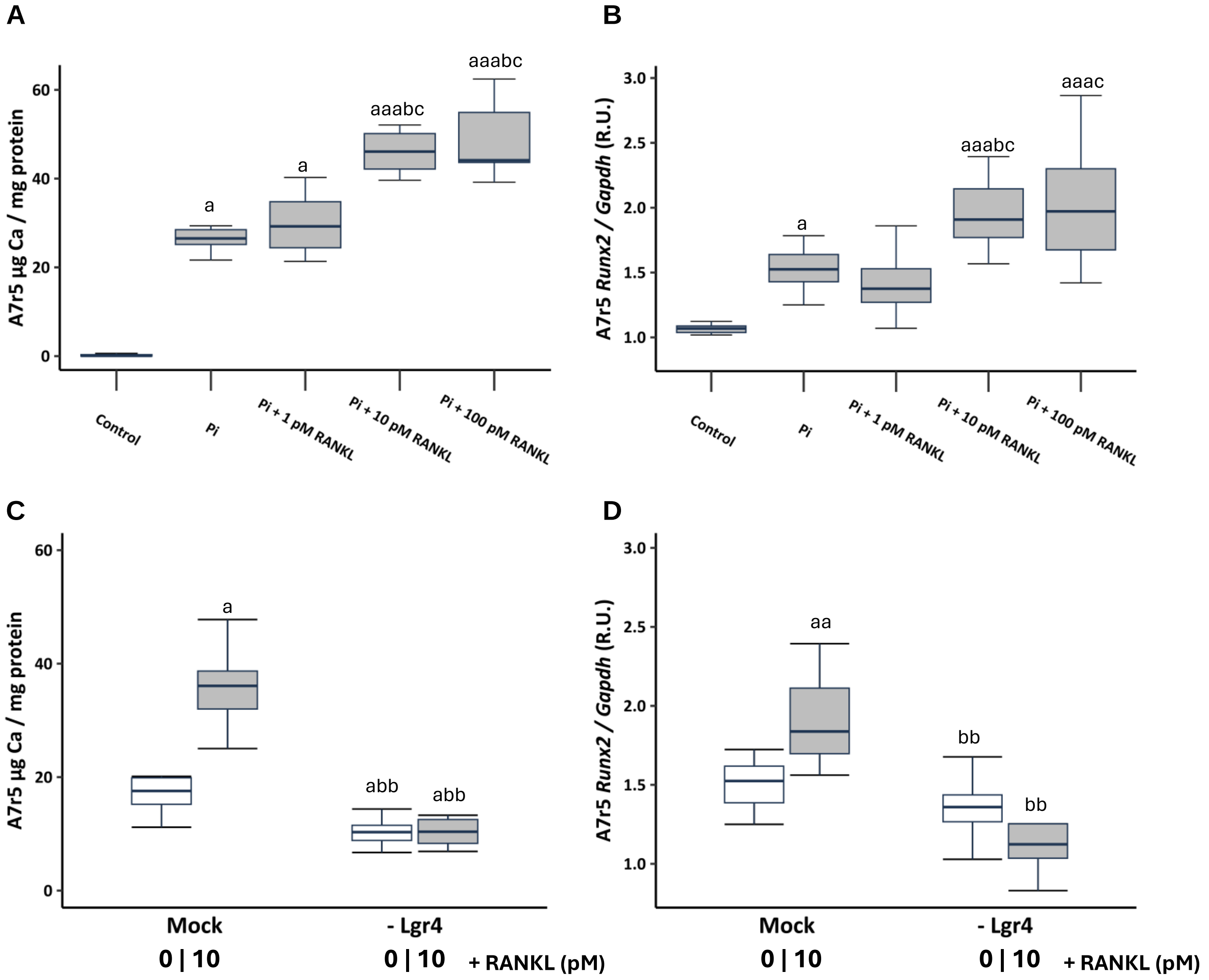

Figure S2: Effect of different soluble RANKL concentrations (1, 10, and 100 pM) on A7r5 vascular smooth muscle cells after 4 days of exposure in calcifying medium (3 mM phosphate, Pi). Ca content determined by *o*-cresolphtalein complexone method (**A**) and *Runx2* gene expression evaluated by qRT-PCR (**B**). $^{a}p < 0.05$, $^{aa}p < 0.01$, $^{aaa}p < 0.001$ versus control (non-calcifying medium, 1 mM phosphate); $^{b}p < 0.05$, $^{bb}p < 0.01$, versus calcifying medium (3 mM phosphate, Pi); $^{c}p < 0.05$, versus calcifying medium (3 mM phosphate) + 1 pM RANKL. Effect of LGR4 silencing on A7r5 vascular smooth muscle cells exposed to soluble RANKL (10 pM) in calcifying medium (3 mM phosphate). Ca content (**C**) and *Runx2* gene expression (**D**). White and grey boxes represent non-addition of RANKL and the addition of 10 pM of RANKL respectively. Mock, scramble transfection control of silencing. $^{a}p < 0.05$, $^{aa}p < 0.01$ versus Mock (0 pM RANKL); $^{bb}p < 0.01$, versus Mock + 10 pM RANKL. Data are presented as median [interquartile range]. R.U., relative units.

Table S1 Effect of high phosphate (3 mM, Pi) and different PTH concentrations (10^{-9}, 10^{-8} and 10^{-7} M) on the differentiation of A7r5 vascular smooth muscle cells after 4 days of exposure The expression levels of *α-actin, Runx2,* and *Osterix* were evaluated by qRT-PCR. Data are presented as median [interquartile range]. R.U., relative units. $^{a}p < 0.05$, $^{aa}p < 0.01$, $^{aaa}p < 0.001$ versus control (non-calcifying medium, 1 mM phosphate); $^{b}p < 0.05$, versus Pi (calcifying medium, 3 mM phosphate).

	Control	Pi	PTH 10^{-9} M	PTH 10^{-8} M	PTH 10^{-7} M	Pi+PTH 10^{-9} M	Pi+PTH 10^{-8} M	P+PTH 10^{-7} M
***α-actin/ Gapdh* (R.U.)**	0.93 [0.84,1.15]	0.52 [0.46, 0.63]^{aaa}	0.82 [0.77, 0.87]	0.82 [0.72, 0.87]	0.80 [0.73, 0.85]^{aaa}	0.61 [0.58, 0.67]^{aaa}	0.60 [0.51, 0.70]^{aaa}	0.50 [0.43, 0.56] ^{aaa}
***Runx2/ Gapdh* (R.U.)**	1.00 [8.66, 1.12]	1.93 [1.73, 2.04] ^{aaa}	1.12 [1.03, 1.19]	1.16 [1.12, 1.27]	1.24 [1.11, 1.41]	1.90 [1.76, 2.09]^{aaa}	2.70 [1.82, 2.17]^{aaa}	2.51 [2.45, 2.6] ^{aaab}
***Osterix/ Gapdh* (R.U.)**	1.02 [0.98, 1.09]	1.48 [1.32, 1.66]	1.03 [0.94, 1.264]	0.88 [0.78, 1.24]	1.01 [0.872, 1.44]	1.47 [1.19, 1.99]	1.65 [1.49, 1.74] ^{aa}	2.46 [1.84, 2.83]^{aaab}

Table S2. Gene expression of vascular smooth muscle cells differentiation markers evaluated in the aortas of control (SHAM) and nephrectomized (NX) rats with (PTX) or without parathyroidectomy, fed normal (NP) or high (HP) phosphorus diet for 18 weeks. The expression levels of *α-actin*, *Runx2*, and *Osterix* were measured using qRT-PCR. The groups are: SHAM NP (Sham operated rats fed normal phosphorus diet), SHAM HP (Sham operated rats fed high phosphorus diet), PTX NX NP (parathyroidectomized and nephrectomized rats fed normal phosphorus diet), PTX NX HP (parathyroidectomized and nephrectomized rats fed high phosphorus diet), NX NP (nephrectomized rats fed normal phosphorus diet), and NX HP (nephrectomized rats fed high phosphorus diet). Data are presented as median [interquartile range]. R.U., relative units. $^{a}p < 0.05$, $^{aa}p < 0.01$, $^{aaa}p < 0.001$ versus SHAM NP; $^{b}p < 0.05$, $^{bb}p < 0.01$, versus SHAM HP; $^{c}p < 0.05$ versus NX NP.

	**SHAM NP**	**SHAM HP**	**PTX NX NP**	**PTX NX HP**	**NX NP**	**NX HP**
***α-actin/ Gapdh* (R.U.)**	0.67 [0.65, 0.69]	0.59 [0.41, 0.72]	0.48 [0.32, 0.66]	0.32 [0.23, 0.39] ^{aa}	0.66 [0.34, 0.73]	0.28 [0.25, 0.37]^{aaabc}
***Runx2/ Gapdh* (R.U.)**	1.22 [0.74, 1.23]	2.30 [1.79, 2.61] ^{a}	2.02 [1.49, 6.15]^{a}	3.14 [1.91, 6.96]^{aa}	3.24 [2.28, 4.17]^{aa}	3.51 [2.37, 5.74]^{aaa}
***Osterix/ Gapdh* (R.U.)**	0.79 [0.54, 1.34]	1.10 [0.66, 2.61]	1.70 [0.94,5.33] ^{a}	6.43 [4.41, 6.97]^{aa}	2.96 [2.43, 5.82] ^{aa}	5.74 [3.88, 9.73]^{aaabb}

Table S3. List of siGENOME SMART pool Lgr4 (Dharmacon™)

siRNA Lgr4	Target sequence
siRNA D-95441-01	CGAAGAACAUAGCCAAAUA
siRNA D-95441-02	GGAACAGGAUUUCUACUAU
siRNA D-95441-03	GAACAAUAUCACCCAGUUA
siRNA D-95441-04	GCGCUAUGCUUACAAUCUA

Table S4. List of Taqman probes used

Gen	Rat reference	Human reference
α-actin	Rn01759928_g1	-
Alp	Rn01516028_m1	-
Gapdh	Rn99999916_s1	Hs99999905_m1
Lgr4	Rn00597924_m1	Hs00173908_m1
Rankl	Rn00589289_m1	Hs00243522_m1
Rspo1	Rn01517010_m1	Hs00543475_m1
Rspo2	Rn01505834_m1	Hs04400416_m1
Rspo3	Rn01537475_m1	Hs00262176_m1
Rspo4	Rn06186317_m1	Hs01382765_m1
Runx2	Rn01512298_m1	Hs00231692_m1
Opg	Rn005634999_m1	Hs00900358_m1
Osx	Rn02769744_s1	-

Table S5. List of abbreviations

ALP	Alkaline Phosphatase
ATCC	American Type Culture Collection
Ca	Calcium
CKD	Chronic Kidney Disease
CRF	Chronic Renal Failure
Ct	Cycle Threshold
DBP	Diastolic Blood pressure
DMEM	Dulbecco's Modified Eagle Medium
DNA	Deoxyribonucleic Acid
ELISA	Enzyme-Linked Immunosorbent Assay
FBS	Fetal Bovine Serum
FGF23	Fibroblast Growth Factor 23
GAPDH	Glyceraldehyde-3-phosphate dehydrogenase
HP	High Phosphorus
IQR	Interquartile Range
KI	Kauppila Index
LGR4	Leucine-rich repeat-containing G-protein-coupled Receptor 4
M	Molar
mM	millimolar
NP	Normal Phosphorus
NX	Nephrectomy
OPG	Osteoprotegerin
P	Phosphorus
Pi	Phosphate
PCR	Polymerase Chain Reaction
pM	picomolar
PTH	Parathormone
PTX	Parathyroidectomy
qRT-PCR	Quantitative Reverse Transcription Polymerase Chain Reaction
RANK	Receptor Activator of Nuclear Factor kappa β
RANKL	RANK ligand
RNA	Ribonucleic Acid
RSPOs	R-spondins
RSPO1	R-spondin 1
RSPO2	R-spondin 2
RSPO3	R-spondin 3
RSPO4	R-spondin 4
R.U.	Relative Units
RUNX2	Runt-related transcription factor (RUNX) family transcription factor 2
SBP	Systolic Blood Pressure
SDS	Sodium Dodecyl Sulfate
siRNA	Small Interfering RNA
VSMCs	Vascular Smooth Muscle Cells
